# Practice variation in anti-epileptic drug use for neonatal hypoxic-ischemic encephalopathy among regional NICUs

**DOI:** 10.1186/s12887-019-1441-7

**Published:** 2019-02-27

**Authors:** Maria L. V. Dizon, Rakesh Rao, Shannon E. Hamrick, Isabella Zaniletti, Robert DiGeronimo, Girija Natarajan, Jeffrey R. Kaiser, John Flibotte, Kyong-Soon Lee, Danielle Smith, Toby Yanowitz, Amit M. Mathur, An N. Massaro

**Affiliations:** 1 Ann & Robert H. Lurie Children’s Hospital of Chicago and Feinberg School of Medicine, Northwestern University, 225 East Chicago Ave, Box 45, Chicago, IL 60611 USA; 2 Washington University, St. Louis, MO USA; 3 Children’s Healthcare of Atlanta, Atlanta, GA USA; 4 Children’s Hospital Association, Overland Park, KS USA; 5 Seattle Children’s Hospital/University of Washington, Seattle, WA USA; 6 Children’s Hospital of Michigan, Detroit, MI USA; 7 Penn State Health Children’s Hospital, Hershey, PA USA; 8 Children’s Hospital of Philadelphia, Philadelphia, PA USA; 9 Hospital for Sick Children, Toronto, ON Canada; 10 Children’s Hospital Colorado, Denver, CO USA; 11 University of Pittsburgh School of Medicine, Pittsburgh, PA USA; 12 Children’s National Health Systems, Washington, DC USA

**Keywords:** Hypoxic-ischemic encephalopathy, Anti-epileptic drugs, Neonatal seizures

## Abstract

**Background:**

While intercenter variation (ICV) in anti-epileptic drug (AED) use in neonates with seizures has been previously reported, variation in AED practices across regional NICUs has not been specifically and systematically evaluated. This is important as these centers typically have multidisciplinary neonatal neurocritical care teams and protocolized approaches to treating conditions such as hypoxic ischemic encephalopathy (HIE), a population at high risk for neonatal seizures. To identify opportunities for quality improvement (QI), we evaluated ICV in AED utilization for neonates with HIE treated with therapeutic hypothermia (TH) across regional NICUs in the US.

**Methods:**

Children’s Hospital Neonatal Database and Pediatric Health Information Systems data were linked for 1658 neonates ≥36 weeks’ gestation, > 1800 g birthweight, with HIE treated with TH, from 20 NICUs, between 2010 and 2016. ICV in AED use was evaluated using a mixed-effect regression model. Rates of AED exposure, duration, prescription at discharge and standardized AED costs per patient were calculated as different measures of utilization.

**Results:**

Ninety-five percent (range: 83–100%) of patients with electrographic seizures, and 26% (0–81%) without electrographic seizures, received AEDs. Phenobarbital was most frequently used (97.6%), followed by levetiracetam (16.9%), phenytoin/fosphenytoin (15.6%) and others (2.4%; oxcarbazepine, topiramate and valproate). There was significant ICV in all measures of AED utilization. Median cost of AEDs per patient was $89.90 (IQR $24.52,$258.58).

**Conclusions:**

Amongst Children’s Hospitals, there is marked ICV in AED utilization for neonatal HIE. Variation was particularly notable for HIE patients without electrographic seizures, indicating that this population may be an appropriate target for QI processes to harmonize neuromonitoring and AED practices across centers.

## Background

Although seizures occur in 26–65% of neonates with hypoxic-ischemic encephalopathy (HIE), it is well known that anti-epileptic drug (AED) management is variable among centers [1–4]. There are several possible reasons for this variability. Neonatal seizures are often subclinical, difficult to detect and cannot be predicted adequately by clinical variables alone [5, 6]. Furthermore, limitations in available resources to detect seizures, as well as a lack of consensus for seizure management among treating neonatologists and child neurologists lead to inconsistent recognition and treatment of neonatal seizures [7, 8]. Continuous electroencephalographic (cEEG) monitoring is therefore recommended in the management of neonates with encephalopathy [9]. However, cEEG is resource intensive and may not be available in all cooling centers. Even when available, factors such as time to application and interpretation may not be uniform across centers. Amplitude-integrated EEG (aEEG) is an alternative form of easily interpretable neuromonitoring that is routinely used in many but not all centers. Finally, the use of selective head cooling for treatment of HIE may temporarily preclude continuous EEG monitoring during therapeutic hypothermia (TH). Detection of subclinical seizures is important because treatment of subclinical seizures reduces seizure burden, and longer duration of seizures is associated with more severe brain injury on MRI and lower performance scores in all domains of the Bayley Scares of Infant Development-III [10, 11].

Variation also exists in the choice of AEDs. Phenobarbital is the first-line AED for treatment of neonatal seizures despite limited evidence to support its use over other agents, [12–14] either for treatment or for seizure prophylaxis [15–17]. Common second-line AEDs for persistent seizures include phenytoin (with similar effectiveness as phenobarbital) [14] and benzodiazepines. More recently, levetiracetam and topiramate are increasingly being used in NICUs as second-line AEDs [8, 18] and are under investigation for potential neuroprotective qualities [19]. Lidocaine has also been described as an AED [14, 20]. Unfortunately, the field has few randomized trials in neonates proving safety or efficacy of one AED over another. A clinical trial of bumetanide as a second-line AED for electrographic seizures not responsive to phenobarbital did not show efficacy but did show the serious side-effect of hearing impairment [21]. The recently completed clinical trial of levetiracetam as first-line therapy for neonatal seizures (NEOLEV2 NCT01720667) reportedly did not show greater efficacy of levetiracetam over phenobarbital (Child Neurology Society Annual Meeting, Chicago, IL, October 16, 2018). Consistent and rational use of these drugs is important as pre-clinical and clinical studies have raised concern regarding AED-associated neurotoxicity in the developing brain, with detrimental effects on neurogenesis, cell proliferation and migration, apoptosis, synaptogenesis and white matter integrity [22–26].

Reducing intercenter variation (ICV) through standardization of care has been demonstrated to improve outcomes across NICU populations [27]. Importantly, several centers have shown that protocol-driven management of neonates at risk for seizures results in improvements in care including diagnosis of seizures [28], decreased phenobarbital levels, progression to status epilepticus, length of hospital stay [29] and discharge on AED [30]. (Improvement in outcomes due to protocolized approaches has been shown in management of other neonatal diseases as well, including congenital diaphragmatic hernia [31] and short bowel syndrome [32]). An important step to improving consistency of care is to understand determinants of variability in AED prescribing practices. Recent studies have reported exposure trends over time and ICV in AED use for neonatal seizures [7, 8, 33, 34]. A consistent message from these reports is the widespread ICV in AED practices, which is not surprising given that prior investigations have evaluated populations of mixed diagnoses and data from various NICUs with different levels of care. Even though neuromonitoring and neuroimaging technology and child neurology specialists are readily available, CHND NICUs do not share standardized treatment protocols. Therefore, we hypothesized that seizure treatment for HIE would vary among the quaternary care Children’s Hospitals in our large consortium. Our objective was to identify sources of ICV in AED utilization with the plan to identify opportunities for quality improvement (QI).

## Methods

Using linked data from the Children’s Hospital Neonatal Database (CHND) and Pediatric Health Information Systems (PHIS) we quantified ICV in the use of AEDs (initiation, selection and duration) and AED cost as another proxy measurement of AED use for neonates with HIE.

### Data sources

CHND prospectively captures detailed clinical data from all infants admitted to 34 participating level IV NICUs [27]. PHIS contains detailed hospital administrative and billing data from > 40 pediatric institutions [35]. Twenty-four CHND sites participate in PHIS. Methods insuring data quality for both databases have been reported [27, 35–38]. CHND and PHIS data were linked at the patient level using unique identifiers unavailable to investigators.

### Study population

CHND was queried to identify neonates born at participating centers between July 2010 and July 2016 with the diagnosis of perinatal HIE according to established criteria [3], treatment with TH, admitted <2 d of life, ≥36 weeks’ gestation and ≥ 1800 g at birth. Neonates were excluded if they had major congenital anomalies or if linkage to PHIS was not possible. The Institutional Review Board at each participating institution approved participation in CHND and associated research studies.

### Data collection

Data regarding antenatal, maternal, birth and delivery characteristics including mode of delivery as well as clinical and demographic data were abstracted according to a CHND manual of operations [27]. Additional detailed neurological data were recorded for neonates with HIE including results of continuous electroencephalographic monitoring (cEEG) within 24 h, amplitude-integrated EEG (aEEG) studies at 24 h and neuroimaging findings on magnetic resonance imaging (MRI). Clinical seizures were also recorded.

### Estimation of AED use

AED use was quantified using four distinct approaches to capture different aspects of use. PHIS Clinical Transaction Classification (CTC) pharmacy codes corresponding to any type of AED were used to quantify frequency, type and duration of AED use per patient. *AED exposure* was defined as ≥1AED CTC code during the initial hospitalization in a given patient. *AED duration* was defined as total number of hospital days with ≥1 AED CTC codes assigned. Neonates were also classified by whether or not they received an *AED at discharge*.

### Cost estimation

Standardized costs were calculated according to a previously described cost master index [35, 39]. Briefly, costs for every billing CTC code were computed and adjusted for wage and price index. All costs were inflated to 2012 dollars and a standardized unit cost for each CTC code was defined as the median cost across all participating hospitals. Standardized costs for all AED-associated CTC codes were calculated per patient. Costs were also calculated for individual AEDs (i.e., phenobarbital, levetiracetam, fosphenytoin/phenytoin, oxcarbazeine, topiramate and valproate). Benzodiazepines (midazolam and lorazepam) were not included in overall models because we could not confirm whether these medications were being used as AEDs or for sedation.

### Data analysis

Study sample size was based on a convenience sample of consecutive admissions of infants meeting inclusion criteria during the study period. Study population characteristics and cost distribution data were described using standard summary statistics after stratifying by presence of seizures noted on EEG (cEEG or aEEG). ICV in AED exposure was evaluated using a logistic regression model, ICV in AED duration was evaluated using a generalized linear model and ICV in AED costs per case was evaluated using a mixed-linear model adjusting for gestational age, sex, electrographic seizures during hospitalization, HIE severity and mortality. Cost data were log transformed to account for the skewed distribution. Data were analyzed using SAS Enterprise Guide 7.1 (SAS Institute Inc., Cary, NC).

## Results

### Study population

**Fig. 1:**
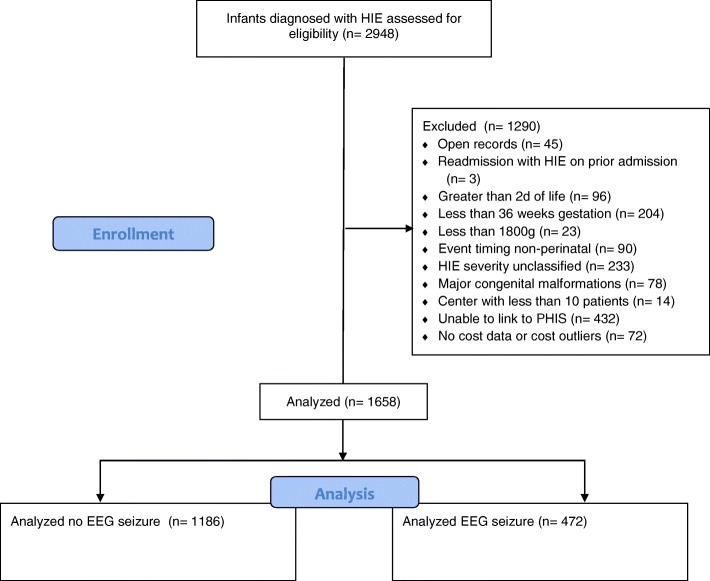
CONSORT flow diagram

Of the 120,601 infants included in the CHND at the time of analysis, we identified 2903 neonates with HIE treated with TH. Neonates admitted at > 2 d of life, < 36 weeks’ gestation, birthweight < 1800 g, with event timing classified as non-perinatal or with major congenital anomalies were excluded (*n* = 727), leaving 2176 neonates. We were able to link 1744 of the 2176 (80%) remaining neonates to their PHIS data. After eliminating additional neonates with systematic errors in PHIS data, we were left with 1658 of 2176 (76%) who met study inclusion criteria (Fig. 1). These neonates were cared for at 20 centers in the US. Median beds per NICU at these centers was 60 (range 28–173). The median number of babies treated with TH per center for the study period was 75 (range 12–187). Each of the centers had a NeuroNICU program and/or the daily involvement of a neurologist. None of the centers provided prophylactic phenobarbital as part of usual practice.

Characteristics of the study population were stratified by presence of clinical or electrographic seizures (Table 1). Ninety-eight percent of all neonates received some form of EEG monitoring (aEEG or cEEG). The exact timing of initiation of cEEG or aEEG monitoring was not known although it was known if studies were done before or after 3d of life; almost all studies were done by 24 h of life. As expected, Apgar scores ≤5 at 5, 10 and 15 min of life, encephalopathy severity, resuscitation in the delivery room (including mechanical ventilation, chest compressions and epinephrine), severe acidosis (pH ≤ 7.00) and use of vasopressors were more frequent in neonates with electrographic seizures. There was no difference in acute perinatal sentinel events [40] between groups with the exception of fetal distress. Encephalopathy grade differed by seizure group. The rate of babies with mild-moderate encephalopathy increased from 60% to over 80% during the study period. Eighteen percent of the cohort (308 of 1658) had mild encephalopathy; of these 5.8% had clinical seizures. The majority underwent total body cooling. We observed a higher rate of seizures in neonates who were selectively head-cooled in contrast with those who received whole body cooling. There was no difference between groups in use of inhaled nitric oxide or extracorporeal membrane oxygenation. Unadjusted rates of mortality were higher and lengths of CHND hospital stay were longer in neonates with electrographic seizures (Table 1). Length of hospital stay included total stay in the CHND hospital (i.e. includes within hospital transfer out of the NICU but not to an outside facility for subacute care).

**Table 1:** Clinical characteristics of study subjects

	All	No Seizure	EEG Seizure	Clinical Seizure	*p*-value
	1658	947	472	239	
Gestational age in weeks Mean (SD)	38.80 (1.48)	38.74 (1.49)	38.97 (1.48)	39 (1.41)	0.010*
Birth weight in grams Median [IQR]	3290 [2940, 3700]	3295 [2940, 3715]	3270 [2890, 3635]	3310 [3000, 3720]	0.206
Female sex (n, %)	721 (43.5)	395 (41.7)	223 (47.3)	103 (43.1)	0.139
Maternal race (n, %)					0.007*
White	996 (60.1)	569 (60.1)	289 (61.2)	138 (57.7)	
Black	350 (21.1)	217 (22.9)	92 (19.5)	41 (17.2)	
Asian	72 (4.3)	43 (4.5)	20 (4.2)	9 (3.8)	
Other	164 (9.9)	73 (7.7)	51 (10.8)	40 (16.7)	
Delivery type (n, %)					0.862
Cesarean	1069 (64.5)	617 (65.2)	301 (63.8)	151 (63.2)	
Vaginal, non-operative	407 (24.6)	223 (23.6)	120 (25.4)	64 (26.8)	
Vaginal, operative	175(10.6)	102 (10.8)	49 (10.4)	24 (10.0)	
Unknown	7 (0.42)	5 (0.53)	2 (0.42)	0(0)	
Apgar score < = 5					
5 min Median [IQR]	3 [1,4]	3 [2,4]	2 [1,4]	2 [1,4]	<0.001*
10 min Median [IQR]	3 [2,5]	4 [2,5]	3 [2,4]	3 [1,4]	<0.001*
15 min Median [IQR]	3 [2,5]	4 [2,5]	3 [2,4]	2 [1,4]	0.012*
DR resuscitation					
ETT/Ventilation (n, %)	1190 (71.8)	631 (66.6)	375 (79.5)	184 (77.0)	<0.001*
Chest compressions (n, %)	688 (41.5)	319 (33.7)	238 (50.4)	131 (54.8)	<0.001*
Epinephrine (n, %)	375 (22.6)	156 (16.5)	144 (30.5)	75 (31.4)	<0.001*
Presenting pH^♯^ Median [IQR]	6.99 [6.82, 7.14]	7.02 [6.88, 7.15]	6.96 [6.80, 7.12]	6.9 [6.80, 7.10]	0.001*
Presenting BD Median [IQR]	16 [11.5, 21]	15 [11.5, 18.8]	17 [12, 22.3]	17.9 [13.3, 24.8]	0.001*
Perinatal sentinel event					
Nuchal cord (n, %)	288 (17.4)	174 (18.4)	79 (16.7)	35 (14.6)	0.36
Cord prolapse (n, %)	54 (3.3)	31 (3.3)	11 (2.3)	12 (5.0)	0.16
Uterine rupture (n, %)	96 (5.8)	46 (4.9)	34 (7.2)	16 (6.7)	0.17
Placental abruption (n, %)	196 (11.8)	99 (10.5)	67 (14.2)	30 (12.6)	0.11
Fetal distress (n, %)	362 (21.8)	232 (24.5)	91 (19.3)	39 (16.3)	0.007*
Continuous inotropes on transport/admission (n, %)	318 (19.2)	150 (15.8)	116 (24.6)	52 (21.8)	< 0.001*
Maternal antenatal conditions					
Chorioamnionitis (n, %)	140 (8.4)	78 (8.24)	49 (10.4)	13 (5.4)	0.08
Diabetes (n, %)	198 (11.9)	126 (13.3)	36 (7.6)	36 (15.1)	0.002*
Encephalopathy severity^+^					<0.001*
Mild (n, %)	308 (18.6)	261 (27.6)	29 (6.1)	18 (7.5)	
Moderate (n, %)	917 (55.3)	560 (59.1)	227 (48.1)	130 (54.4)	
Severe (n, %)	433 (26.1)	126 (13.3)	216 (45.8)	91 (38.1)	
Type of cooling					<0.001*
Head (n, %)	337 (20.3)	121 (12.8)	153 (32.4)	63 (26.4)	
Whole body (n, %)	1326 (80.0)	834 (88.1)	320 (67.8)	172 (72.0)	
ECMO	57 (3.4)	37 (3.9)	10 (2.1)	10 (4.2)	0.17
iNO	334 (20.14)	182 (19.2)	90 (19.07)	62 (25.9)	0.05
Pre-discharge mortality (n, %)	223 (13.5)	85 (9.0)	93 (19.7)	45 (18.8)	<0.001*
Survivors hospital LOS Median [IQR]	13 [10, 22]	11 [8, 18]	15 [10, 25.5]	14 [9, 26]	<0.001*
Treated with AED (n, %)	757 (45.6)	133 (11.9)	447 (94.7)	197 (82.4)	<0.001*
Discharged on AED (n, %)	261 (20.1)	11 (1.42)	196 (56.3)	54 (30.7)	<0.001*

*Abbreviations:* *EEG* electroencephalographic, *IQR* interquartile range, *DR* delivery room, *ETT* endotracheal tube, *ECMO* extracorporeal membrane oxygenation, *iNO* inhaled nitric oxide, *LOS* length of stay, *AED* anti-epileptic drug

^♯^Presenting pH = worst umbilical cord gas or arterial blood gas within 1 h of life if cord gas not available

^+^VON or NICHD definitions of HIE were used, depending on each site’s practice; for the NICHD definition, infants with mild encephalopathy on Sarnat exam and seizures qualify for TH

^*^p-value <=0.05

Four hundred seventy-two of 1658 (28.5%) neonates included in the study had seizures noted on cEEG or aEEG at anytime during the first 24 h of admission; cEEGs were used in over two-thirds (1131/1658) and aEEG in nearly one-third (494/1658) of neonates. Not surprisingly, the rate of cEEG monitoring was lower in the selectively head-cooled neonates (only 23% received cEEG within the first 24 h of admission compared to 81% for whole body-cooled neonates; 47% of head-cooled neonates received aEEG compared to 27% for whole body-cooled). Status epilepticus was noted in 2% of all patients (*n* = 27) or 6% of patients with electrographic seizures. Neonates with seizures on cEEG were more likely to have an abnormal background reported at 24 h (Table 2). In contrast, clinical seizures that were not present electrographically were observed in 239 of 1186 (20%) neonates (Table 1). Of these, 200 occurred at or before 3d of life and 39 occurred after 3d of life. Interestingly, 5.8% of cases of mild encephalopathy had clinical seizures (1.1% of the entire cohort) and 9.4% had EEG seizures (1.8% of the entire cohort). On neuroimaging, MRI was completed in 1450 (87%) of neonates and was more often abnormal in neonates with electrographic seizures, with a higher incidence of hemorrhage, stroke, white matter injury and injury to cortex or deep grey nuclei (Table 2).

**Table 2:** Neurophysiologic and MRI findings of study subjects

	All	No Seizure	EEG Seizure	Clinical Seizure	*p*-value
	1658	947	472	239	
aEEG at 24 h^♯^ (n,%)	494 (29.8)	259 (27.4)	141 (29.9)	94 (39.3)	0.001*
Normal	82 (16.6)	69 (26.6)	6 (4.3)	7 (7.5)	<0.001*
Moderately abnormal	223(45.1)	119 (46.0)	76 (53.9)	28 (29.8)	
Severely abnormal	86 (17.4)	25 (9.7)	37 (26.2)	24 (25.5)	
Unknown	103(20.9)	46 (17.8)	22 (15.6)	35 (37.2)	
cEEG within 24 h^♯^ (n,%)	1131(68.2)	707 (74.7)	310 (65.7)	238 (99.6)	<0.001
Short	135 (11.9)	79 (11.2)	28 (9.0)	28 (24.6)	<0.001
Continuous	95 (8.4)	54 (7.6)	24 (7.7)	17 (14.9)	
Video	901 (79.7)	574 (81.2)	258 (83.2)	69 (60.53)	
Diagnosis type					
Normal	172 (15.2)	148 (20.9)	15 (4.8)	9 (7.9)	<0.001*
Abnormal	959 (84.8)	559 (79.1)	295 (95.2)	105 (92.1)	
Background					
Burst suppression	123 (10.9)	27 (3.8)	82 (26.5)	14 (12.3)	<0.001*
Continuous (Normal)	233 (20.6)	195 (27.6)	21 (6.8)	17 (14.9)	
Discontinuous	620 (54.8)	410 (58.0)	157 (50.7)	53 (46.5)	
Isoelectric / Flat	103 (9.1)	44 (6.2)	34 (11.0)	25 (21.9)	
Stated unknown	52 (4.6)	31 (4.4)	16 (5.2)	5 (4.4)	
MRI findings^+^ (n,%)	1450	831	415	204	
Hemorrhage	303 (20.9)	166 (20.0)	103 (24.8)	34 (16.7)	0.039*
Stroke	107 (7.4)	46 (5.5)	45 (10.8)	16 (7.8)	0.003*
White matter injury	236 (16.3)	98 (11.8)	98 (23.6)	40 (19.6)	<0.001*
Deep grey matter injury	284 (19.6)	84 (10.1)	147 (35.4)	53 (26.0)	<0.001*
Cortical injury	183 (12.6)	41 (4.4)	115 (27.7)	27 (13.2)	<0.001*
Normal	524 (36.1)	400 (48.1)	72 (17.4)	52 (25.5)	<0.001*

*Abbreviations:* *EEG* electroencephalographic, *MRI* magnetic resonance imaging

^♯^Based on patients for whom aEEG or cEEG data were available; some patients received both aEEG and full EEG (cEEG); aEEG was not consistently displayed on full EEG; aEEG reflects cerebral function monitor output

^+^Based on patients for whom MRI findings were available

^*^p-value <=0.05

### AED selection

**Fig. 2:**
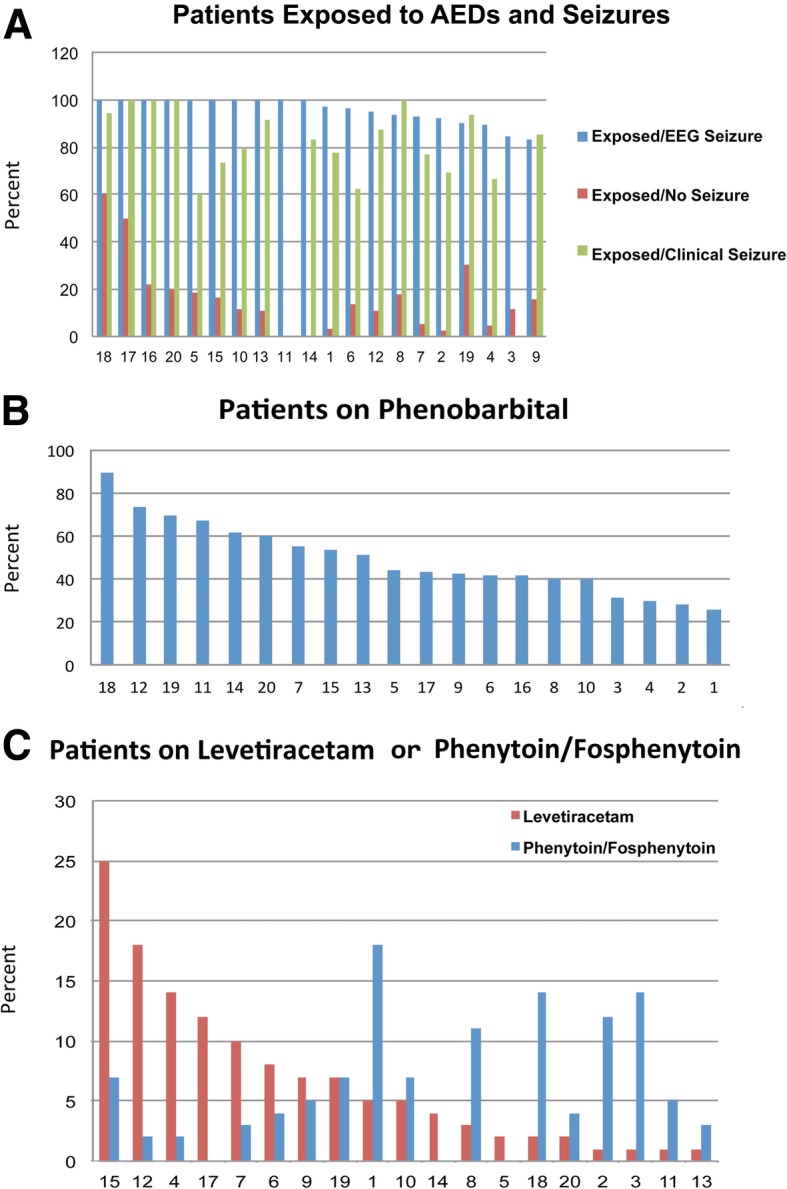
AED exposure by center. **a** Rate of exposure of patients to any AEDs with and without electrographic seizures and with clinical seizures by center. **b** Exposure to phenobarbital by center. **c** Exposure to levetiracetam and phenytoin/fosphenytoin by center

Among patients receiving AEDs with the exclusion of midazolam, lorazepam and clonazepam (*n* = 757), phenobarbital was used most frequently (97.6%), followed by levetiracetam (16.9%), fosphenytoin/phenytoin (15.6%) and others (2.5%; oxcarbazepine, topiramate, valproate) (Table 3). Unadjusted ICV in patient exposure to phenobarbital (Fig. 2b), levetiracetam and phenytoin/fosphenytoin (Fig. 2c) across 20 centers was striking. Frequency of exposure to levetiracetam and fosphenytoin/phenytoin appeared inversely related to each other by center. Two hundred and ninety-five (39%) of patients received only 1 AED, whereas 250 (33%) received 2 and 212 (29%) received 3 or more AEDs. Phenobarbital was the first-line AED throughout the entire study period. The most common second drug changed at the end of the study from fosphenytoin/phenytoin to levetiracetam (Fig. 4g). Interestingly, 10 patients received levetiracetam only. Of note, benzodiazepines were given to 95% of patients.

**Table 3:** Unadjusted AED costs per patient who received AEDs

Category	All		No Seizure		EEG Seizure		Clinical Seizure	
	n	median [IQR] cost	n	median [IQR] cost	n	median [IQR] cost	n	median [IQR] cost
All AEDs	1252	$89.90 [24.52, 265.84]	563	$34.13 [11.38, 100.76]	464	$229.39 [93.73, 511.49]	255	$101.15 [38.91, 209.57]
Phenobarbital	739	$96.96 [35.53, 206.04]	107	$32.32 [13.81, 97.86]	437	$129.79 [59.86, 262.06]	195	$64.64 [24.14, 142.23]
Phenytoin/Fosphenytoin	118	$67.71 [15.46, 238.53]	3	$36.85 [29.78, 259.06]	97	$69.59 [15.46, 238.53]	18	$70.25 [25.91, 123.94]
Levetiracetam	128	$197.62 [59.25, 453.18]	10	$69.82 [13.84, 80.51]	98	$240.09 [105.10, 534.20]	20	$53.39 [21.48, 163.88]
Benzodiazapines	1010	$34.13 [11.38, 91.02]	525	$26.30 [11.37, 82.54]	328	$45.51 [17.33, 102.39]	157	$34.13 [15.39, 91.07]
Other (oxcarbazepine, gabapentin, topiramate, valproate)	19	$102.94 [26.49, 354.27]	3	$19.18 [6.62, 56.29]	15	$134.22 [51.21, 379.45]	1	$26.49 [26.49, 26.49]

*Abbreviations:* *AED* anti-epileptic drug, *EEG* electroencephalographic, *IQR* interquartile range

### AED use in neonates with HIE treated with TH

**Fig. 3:**
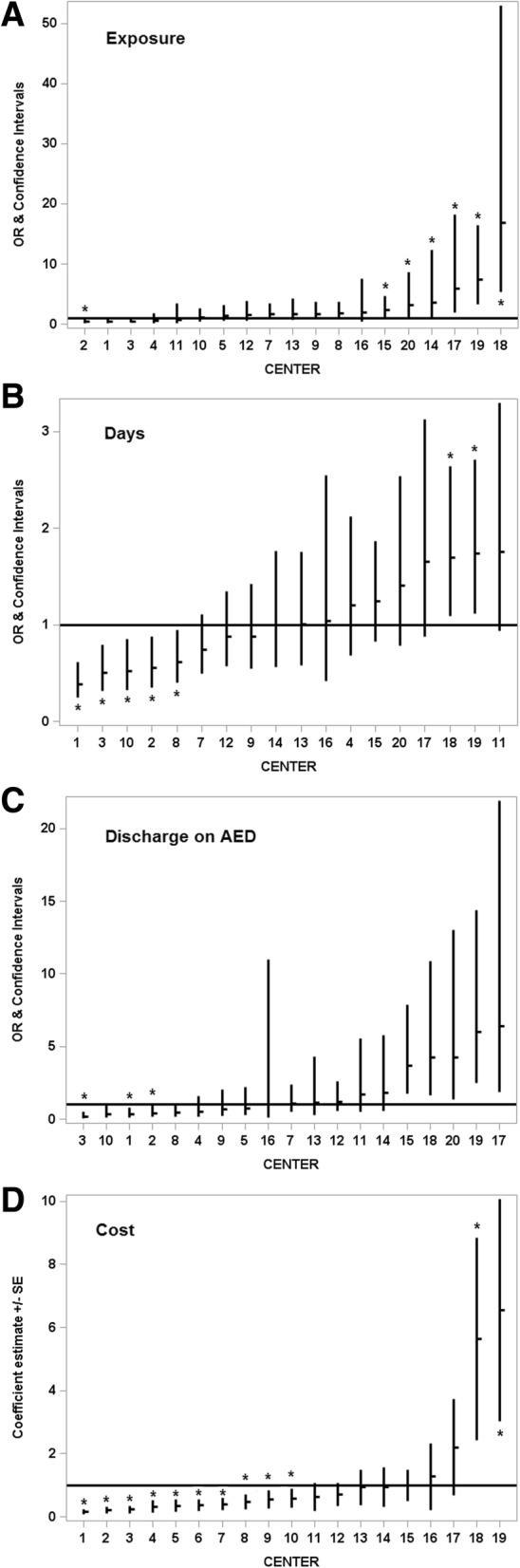
ICV in AED use and cost. OR (odds ratio). *p < 0.05. **a** Any AED exposure by center. **b** Duration of AED exposure by center. **c** Discharge on AED by center. **d** Cost of AED use by center

AEDs were given in 45% of patients overall. Frequencies of AED exposure stratified by the presence of electrographic seizures are shown in Fig. 2a. In patients with electrographic seizures, AED exposure was nearly universal (95%, range 83–100% across centers). Surprisingly, a significant proportion of neonates (26%, range 0–81% across centers) who received AED did not have seizures captured on any type of EEG (Fig. 2a), and in only one center (center 11) no neonate without EEG seizures received an AED. In a logistic regression model adjusting for gestational age, sex, electrographic seizures, status epilepticus, HIE severity and mortality, AED exposure differed significantly across centers (*p* < 0.001) (Fig. 3a). The magnitude of adjusted differences between centers for any AED exposure was estimated as high as 15-fold. Likewise, AED duration (Fig. 3b), evaluated with a generalized linear model, also differed significantly across centers after adjusting for gestational age, sex, electrographic seizures, status epilepticus, HIE severity, mortality and length of stay (*p* < 0.001). Days of exposure to AEDs ranged between 0.5 fold to 1.5-fold adjusted differences. As expected, neonates with electrographic seizures were more frequently discharged on AEDs as compared to those without (56% vs. 6.9%) (Table 1). After adjustment, results from logistic regression show a significant difference across centers in AED use at discharge (*p* < 0.001), as much as 6-fold (Fig. 3c).

### Costs associated with AED use

The median total unadjusted AED cost per patient who received AEDs was $89.90 (IQR $24.52, $258.58). Despite similar frequency of use, costs per patient for levetiracetam were over 2.9 times the costs associated with fosphenytoin/phenytoin and twice the cost of phenobarbital (Table 3). In a mixed-effect linear regression model adjusting for gestational age, sex, electrographic seizures, status epilepticus, HIE severity and mortality, AED cost differed significantly across centers (*p* < 0.001), ranging from 0.5-fold to > 3-fold (Fig. 3d).

### Practice changes over time

**Fig. 4:**
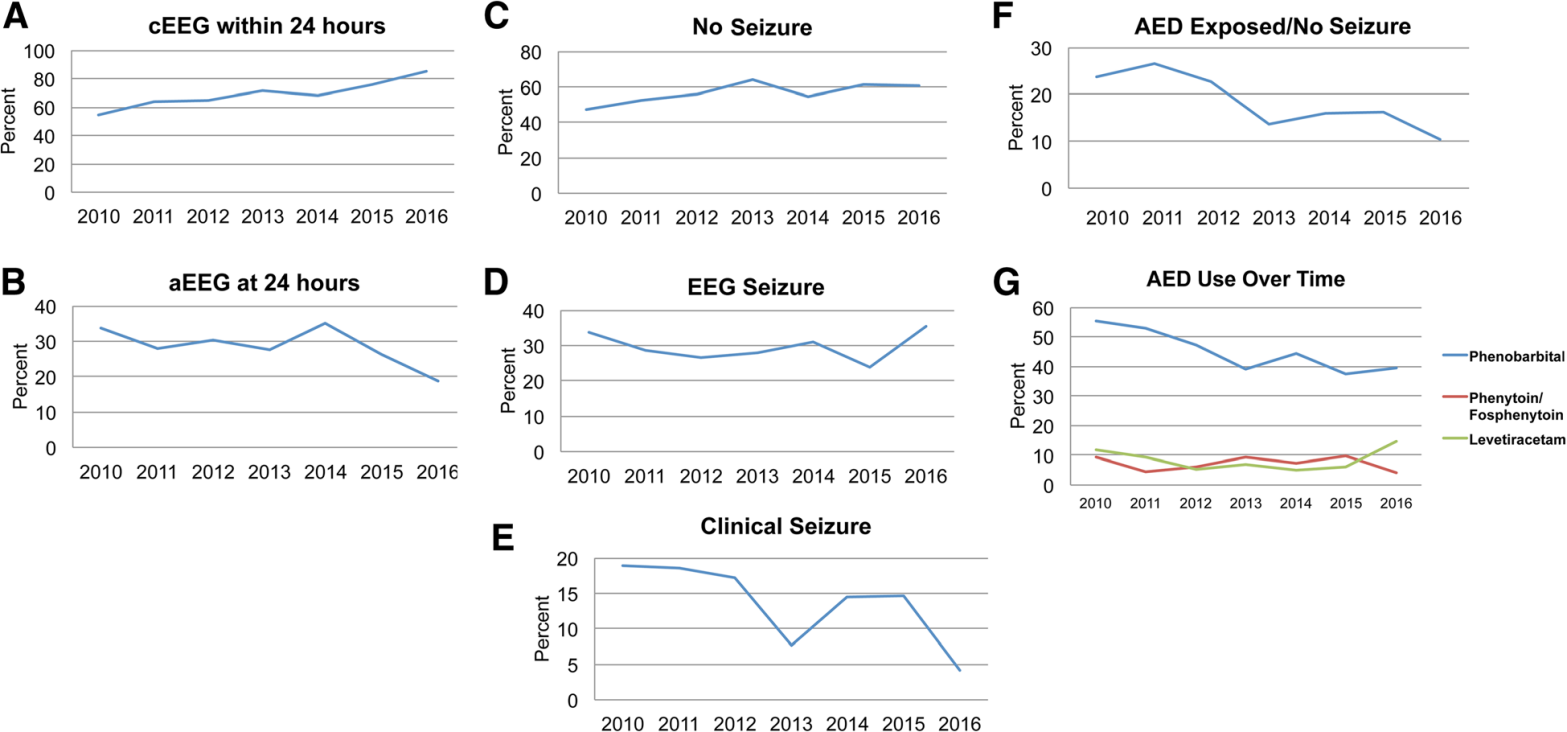
**a** cEEG within 24 h by year. **b** aEEG at 24 h by year. **c** No Seizure by year. **d** EEG Seizure by year. **e** Clinical Seizure by year. **f** AED Exposed/No EEG Seizure by year. **g** AED Use Over Time by year

Rates of cEEG use were 54% at the beginning of the study (2010) versus 64% for 2012, after publication of American Clinical Neurophysiology Society (ACNS) guidelines for EEGs in neonates [9], and 85% at the end of the study (2016) (Fig. 4a). Rates of aEEG use were lower than for cEEG throughout the study, highest at 34% at the beginning of the study with rates of only 19% at the end of the study (Fig. 4b). Proportion of infants diagnosed with EEG seizures remained relatively stable despite an increase in cEEG use (Fig. 4d). By contrast, proportion of infants with clinical seizures only decreased over time (Fig. 4e). There was a similar decrease in infants who received AEDs when no seizures were detected electrographically, from a peak of 27% in 2011 to a low of 10% in 2016 (Fig. 4f). Finally, we looked at rates of individual AEDs by year and observed a decrease in phenobarbital use after 2011, from a peak of 56% to a low of 38% in 2015 (Fig. 4g). We also observed that rates of levetiracetam use surpassed rates of fosphenytoin/phenytoin in 2016 (Fig. 4g).

## Discussion

The purpose of this project was to evaluate the variability that exists across regional NICUs caring for a large burden of neonates with HIE in the US, in order to inform a future QI intervention [41]. In a contemporary cohort of neonates with HIE treated with TH at 20 US regional NICUs, we observed significant ICV in AED utilization. We looked at utilization from a number of different perspectives including selection, any AED exposure, duration of exposure, discharge on AEDs and AED cost as another proxy for utilization. This study of our very large consortium highlighted unwarranted variation [42] in the management of AEDs in HIE, particularly in neonates without electrographic evidence of seizures. This work therefore supports a future QI collaborative across the CHND consortium targeting neonates with HIE who do not have electrographic evidence of seizures. It is important to note that CHND NICUs do not share standardized treatment protocols although all centers have similar levels of care and availability of specialty services. Although best practices have been designated by the state of California (https://www.dhcs.ca.gov/services/ccs/Documents/ccsnl061116.pdf), only 2 California sites were involved in this study and more globally accepted guidelines are not available. Nonetheless, all CHND centers involved in this study met recommendations by the American Academy of Pediatrics Committee on Fetus and Newborn for centers that provide TH, including level III or higher NICU care, neurologic consultation, neuromonitoring with aEEG or cEEG, neuroimaging by MRI, systems for monitoring longitudinal neurodevelopmental outcome, training programs and infrastructure including written protocols and monitoring of outcomes as well as outreach to community hospitals [43].

Despite ACNS guidelines for EEGs in neonates [9], significant variability exists in the application of cEEG for seizure detection/monitoring. We did observe a dramatic increase in use of cEEG overall following publication of the guidelines mid-study in 2011. A decrease in use of aEEG use toward the end of the study period may have been related to discontinued sales of the selective head-cooling device in the US. Although 98% of all neonates in our study received some form of neuro-monitoring (cEEG or aEEG or both), it is possible that our seizure rates are underestimated in those who did not receive monitoring, those who received delayed monitoring or those that received aEEG alone, given the superior sensitivity of cEEG for seizure detection (particularly for seizures that are brief, infrequent or of low amplitude, or not central or parietal [44]). The incidence of seizures detected by EEG in our cohort was 28%, lower than for the CoolCap (61% detected by aEEG) [45], TOBY (54% detected by aEEG) [46] and NICHD hypothermia trials (46% clinical seizures) [47]. Details regarding exact timing of seizure detection and EEG acquisition in relation to AED administration were not available, although it is known that the majority of seizures in HIE occur in the first 24–48 h of life [48, 49]. Status epilepticus rates were lower than expected [2] and may be related to the application of TH to mild HIE cases in real practice. That some clinical seizures occurred in the absence of electrographic seizures might be explained by the following scenarios: clinical movements might not be due to epileptiform activity; seizures noted prior to initiation of cEEG might have spontaneously resolved or resolved following AED given; the threshold to treat clinical seizures during TH might be higher if patients are not on cEEG or aEEG for the entire period of TH and rewarming; even if they were, cEEG reading might not be immediately available. We observed relatively low rates of clinical seizures but a rate of EEG seizures of nearly 10% in cases of mild encephalopathy who were cooled. For these cases, we speculate that clinical or EEG seizures might have been noted after initial assignment of severity category without reassignment to the moderate category after seizures were noted. Our data reinforce that cEEG or aEEG should be obtained in all mild cases of encephalopathy as EEG seizures would indicate that the eligibility for TH had been met.

Consistent with AED selection in other studies [8, 33, 34], we observed a similar predominance of phenobarbital use and a higher frequency of levetiracetam compared to phenytoin/fosphenytoin use. We examined levetiracetam use by year and found an increase in levetiracetam over fosphenytoin/phenytoin in the final year of the study. The apparent inverse relationship of levetiracetam and fosphenytoin/phenytoin use suggests that preferential use of these second-line medications varies by center practice; alternative explanations include fosphenytoin shortages as well as the development of an intravenous formulation of levetiracetam. AED costs per patient were highest for levetiracetam, 2.9-fold greater than fosphenytoin/phenytoin, and cost considerations may drive AED choice for some providers. On the other hand, levetiracetam may be preferred by some providers because of its association with decreased respiratory depression.

Although previous studies have shown ICV in AED utilization, given that the NICUs in our consortium are all Level IV, we were nonetheless somewhat surprised to find the magnitude of ICV that we observed. One study that included some of the same referral centers, observed similar ICV in continuation of AEDs at discharge for neonatal seizures of all etiologies. After univariable analysis adjusting for electrographically confirmed seizures, status epilepticus, seizures refractory to the initial loading dose of AED and abnormal neurological exam at discharge, only study site and seizure etiology remained significantly associated with discharge on AEDs. With regards to seizures specifically associated with HIE, this study’s overall rate of discharge on AEDs was 57%, similar to the 56% that we observed in cases of HIE with electrographically confirmed seizures. Treatment duration differences were implied in this study but not directly reported [8].

Frequency of AED at discharge was center-dependent in our study as well, suggesting that physician/center practice drives the decision to continue AEDs. In our study, over half of neonates with electrographic seizures and 7% of neonates without electrographic seizures were discharged on AEDs. Stated otherwise, if a neonate ever received an AED, that neonate had a 1 in 3 chance of being discharged on an AED. This variation is important because, although neonates with HIE, and particularly those with seizures, are at increased risk for later epilepsy [50, 51], emerging evidence suggests that discharge on an AED might not be indicated in all neonates with acute seizures after HIE [52]. It is well recognized that prolonged use of most AEDs is associated with neuronal apoptosis and neurodevelopmental delays [26, 53]. This added risk is even less acceptable for neonates who have never demonstrated seizures by EEG. Unlike previous studies, we showed ICV in other measures of AED utilization, including any exposure and duration of exposure and cost.

We were surprised to find that a high proportion of neonates without seizures confirmed by EEG received AEDs, many through discharge. This may partly reflect AED use for clinical seizures not confirmed electrographically, and may occur more frequently when EEG is not immediately obtainable as not all centers have 24/7 EEG technician and neurophysiologist capabilities. High rates of AED use in neonates without electrographic seizures, as high as 60% at one center, might also reflect attempts at neuroprotection or seizure prophylaxis by some sites. A recent Cochrane Database meta-analysis did not support the use of prophylactic barbiturates for perinatal asphyxia because, although this practice seemed to reduce seizures, it did not reduce mortality or neurodevelopmental impairment [17]. Our data suggests a need to identify sites that use AEDs for neuroprotection or seizure prophylaxis and to stop this practice.

That a small proportion of neonates with electrographic seizures did not receive AEDs during their hospitalization is also surprising. As our data reflects only medications received at CHND hospitals, it is possible that these neonates received AEDs at the referral hospital that were not continued upon admission to the CHND NICU. It is also possible that limited real-time availability of neurophysiologists across centers may be associated with delayed EEG interpretation and reporting, so that some seizures clinically resolved by the time of recognition on EEG would not lead to AED initiation. Finally, although benzodiazepines are often used to treat intractable seizures or status epilepticus, we did not report the use of benzodiazepines that may have been used to treat seizures; given the nature of the registry, we were unable to confirm whether benzodiazepines were given for seizures or for sedation. The use of AEDs without EEG evidence of seizures offers an opportunity for intervention and change in practice(s).

The major strength of our study was the linkage of clinical data with PHIS data which enabled us to evaluate utilization and cost of AEDs over the course of hospitalization in neonates with HIE. Although a previous study used PHIS data to evaluate AED use, its subjects had neonatal seizures due to various etiologies and were hospitalized during an epoch when TH was not yet standard of care and costs were not evaluated [4]. As TH has led to centralization of care of neonates with HIE to regional NICUs, describing practice variation in this setting is important. Indeed, not all centers that provide TH provide related services such as cEEG or aEEG [54]. We capitalized on detailed clinical information from CHND not available from PHIS alone that allowed us to observe that AED use was significantly affected by gestational age, HIE severity, EEG seizures and mortality, in contrast to the previous study [4]. After controlling for these clinical covariates, ICV in AED use for neonates with HIE persisted.

Another major strength of our investigation was that we only studied neonates with HIE, the most common etiology of neonatal seizures in the current era, who were cared for at regional NICUs. By contrast, prior studies have compared dissimilar groups such as preterm infants or infants with central nervous system disease [4, 33]. Similarly, prior survey and registry-based studies have evaluated data from various NICUs where availability of neurodiagnostic studies (MRI, EEG, etc.) and child neurology specialists may contribute to variations in care [7, 8]. Our study included only regional NICUs meeting criteria for participation in the CNHD [27], and thus highlights the true conundrum of unexplained practice variation with regards to AED use in HIE.

Our study has some limitations. Referral biases exist because some neonates may have died prior to referral to the CHND NICU. Coding differences in AED use may exist between centers despite electronic acquisition of data but processes are in place to insure quality [27]. Unfortunately, we were also unable to link EEG findings temporally to AED initiation and discontinuation. Likewise, details regarding timing of seizure detection and EEG performance in relation to discharge were not available, although it is known that the majority of seizures in HIE occur within the first 24–48 h of life [48, 49]. Developmental outcomes and detailed seizure information is not presently available in CHND. Additionally, given that this study only involved care in regional referral sites, our findings may not be generalizable to community hospitals.

Interestingly, we observed a significantly higher unadjusted rate of seizures in neonates who were selectively head-cooled in contrast with those who received whole body cooling (Table 1). We speculate that delay in obtaining cEEG may result in delay in treatment and a higher rate of seizures at first cEEG. This observation warrants further study given the relatively small number of infants who received selective head cooling, multiple comparisons and unadjusted rates.

## Conclusions

Significant variation exists in AED utilization in neonates with HIE treated with TH within our Children’s Hospital regional NICUs. This data indicates a multicenter QI project within the CHND is in order, with the goal of increasing timely neuromonitoring and eliminating exposure to AEDs without proof of EEG seizures. We believe the rate of exposure to AEDs without EEG seizures should approach 0%. Specific practices to be targeted in our initial QI project will include: 1) observation or use of lorazepam for clinical seizure without EEG confirmation, 2) cEEG or aEEG on admission for all neonates transported for TH (metrics will also include time from admission to placement of cEEG or aEEG), 3) cEEG or aEEG confirmation of seizures prior to phenobarbital, and 4) time from cEEG or aEEG confirmation of seizures to infusion of phenobarbital. Such an effort will improve adherence to evidence-based practices within member hospitals of the CHND.

## Abbreviations

AED: anti-epileptic drug
aEEG: amplitude-integrated EEG
cEEG: continuous electroencephalographic monitoring
CHND: Children’s Hospital Neonatal Database
CTC: Clinical Transaction Classification
HIE: hypoxic ischemic encephalopathy
ICV: intercenter variation
MRI: magnetic resonance imaging
PHIS: Pediatric Health Information Systems
QI: quality improvement
TH: therapeutic hypothermia
